# Supplementary figures and images for: Variation in the geometry of concept manifolds across human visual cortex
Source: PLoS Comput Biol. 2025 Sep 12;21(9):e1013416. doi: 10.1371/journal.pcbi.1013416 (PMC12445742; doi:10.1371/journal.pcbi.1013416)

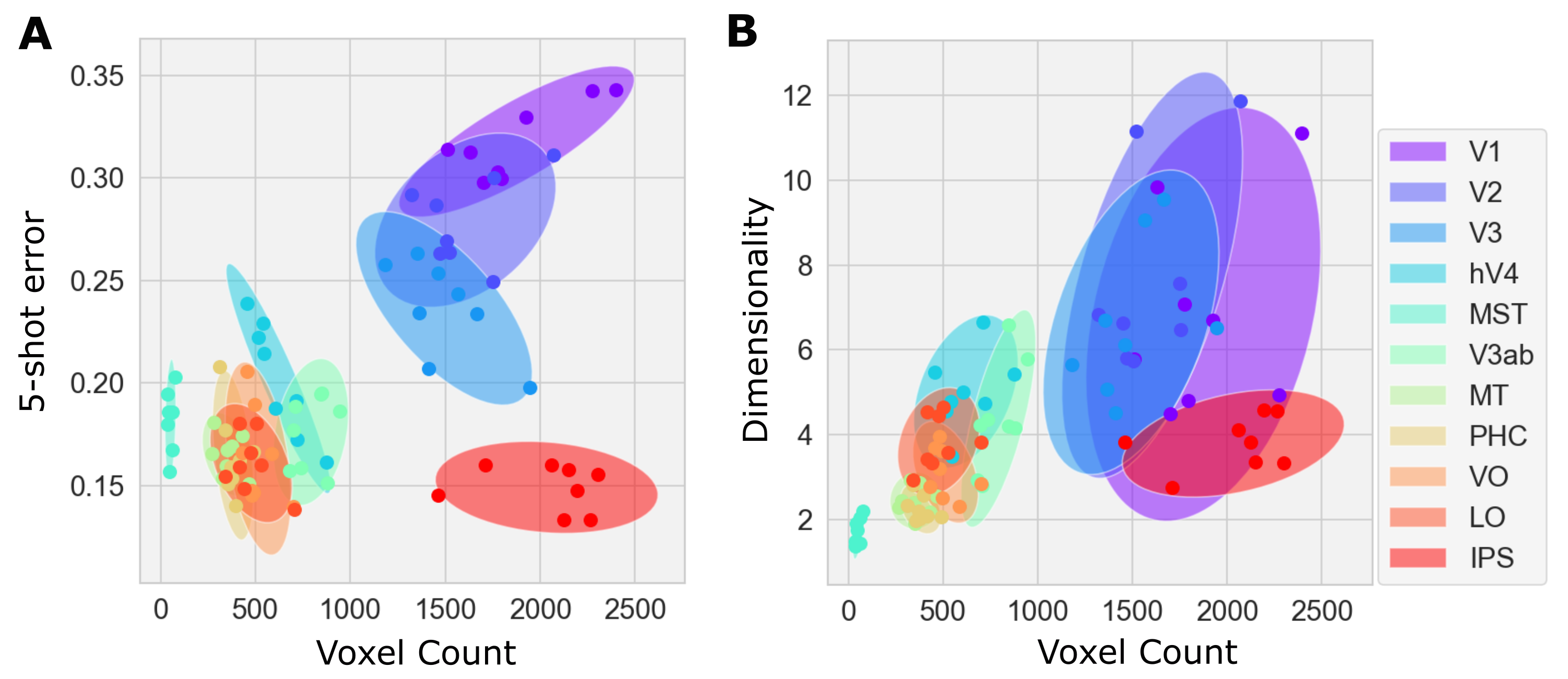

Supplement: S1 Fig — The point correspond to the estimate of the 8 subjects and the ellipses capture 2 std. dev. around the subject average. There is a tendency for ROI higher in the visual hierarchy to have smaller number of constituent voxels. Could this fact alone explain the change in few-shot accuracy and dimensionality? It appears this is not the case since at least one higher ROI, IPS, has constituent voxel count on the same order as early visual areas but nevertheless has vastly superior (inferior) few-shot accuracy (Dimensionality). (TIF) [file pcbi.1013416.s001.tif]

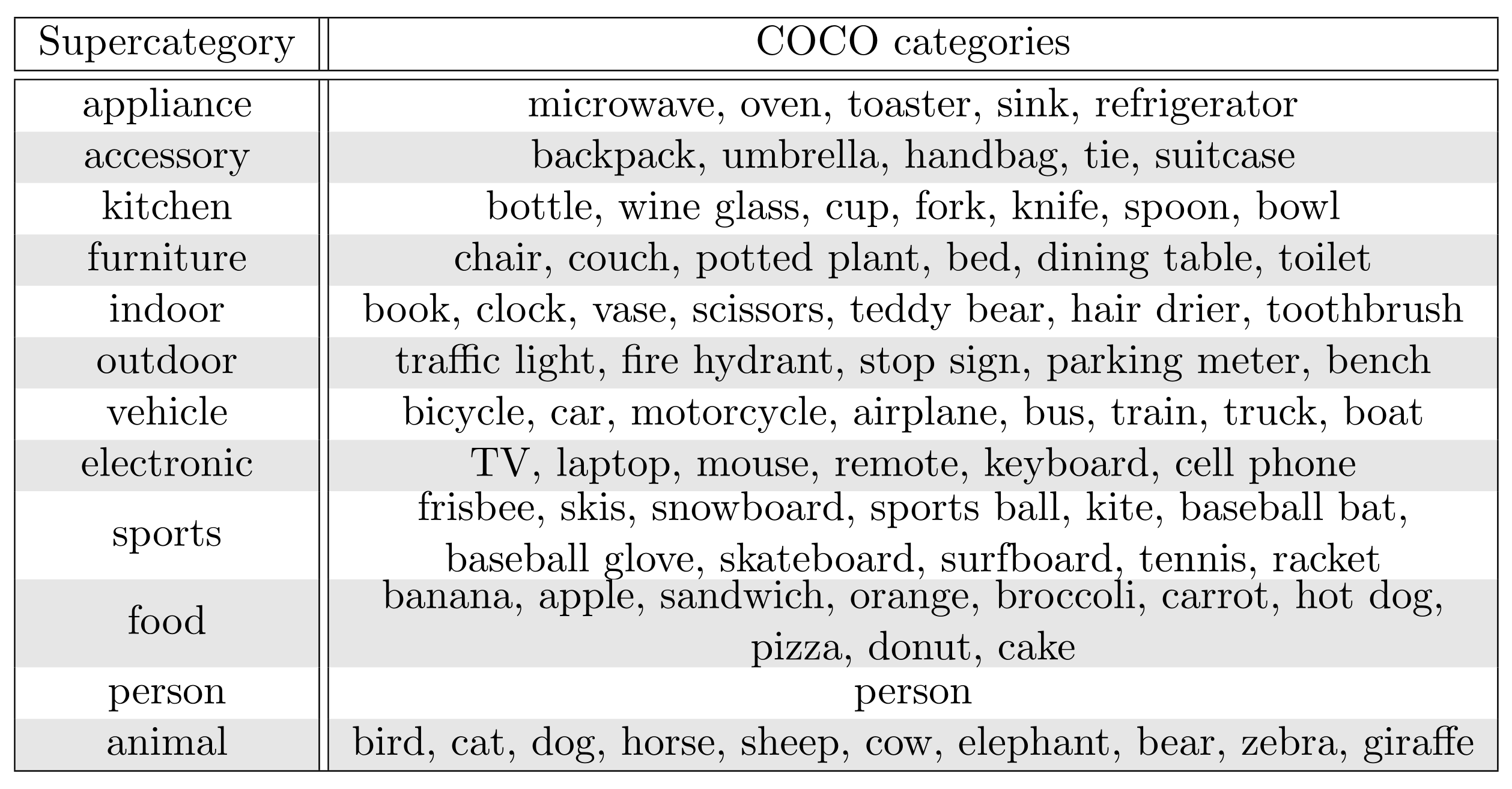

Supplement: S2 Fig — An image is deemed an examplar of a supercategory manifold if some of its pixels belong to an object of one of its constituent objects. Example images where the object belonging to the supercategory have been highlighted with a white surrounding and the remaining pixels have been shaded out slightly to emphasize the pixels determining the supercategory attribution can be found at https://github.com/styvesg/nsd_manifolds. (TIF) [file pcbi.1013416.s002.tif]

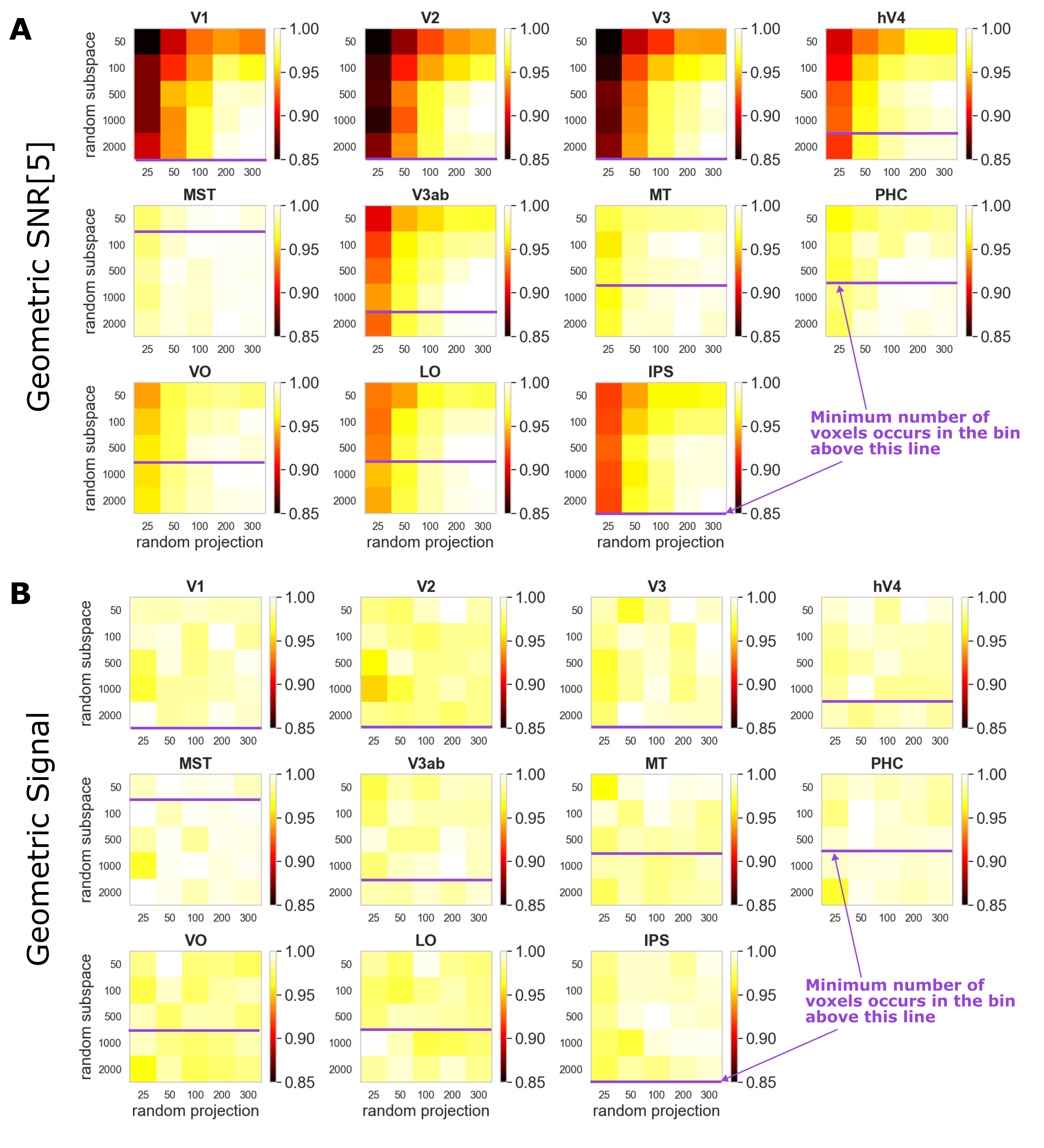

Supplement: S3 Fig — We show the convergence of the geometric estimate as a function of two of the representation embedding hyperparameters: the random subspace dimension (i.e., the number of randomly selected voxels) and the random projection dimension (i.e., the number of random dimensions onto which activity patterns are projected). A) Each panel show the fraction of the estimate of Geometric SNR [5] relative to its maximum in various regions. B) Each panel show the fraction of the estimate of Geometric Signal relative to its maximum in various regions. In both cases, the purple lines provide an estimate of where the random subspace dimension become greater than the number of voxels in that ROI. If that dimension is greater than the number of voxels, then all voxels are always selected. In the main figures of the paper, we used all available voxels in each brain ROI; for networks, the random subspace dimension was fixed at 25,000. (TIF) [file pcbi.1013416.s003.tif]

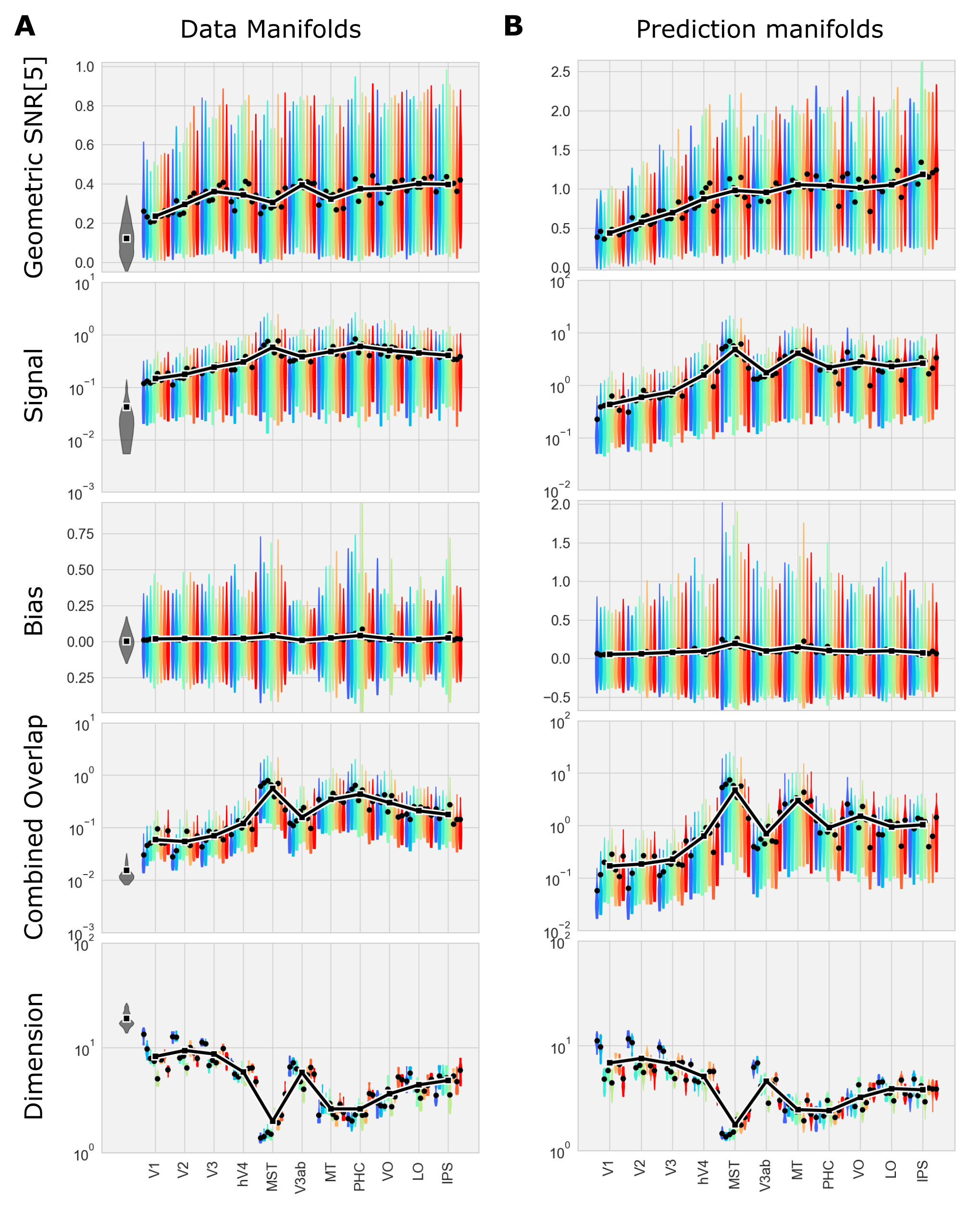

Supplement: S4 Fig — We notice the overall reduction in SNR, Signal and, counter-intuitively, Overlap, while Dimensionality increases for manifold geometry estimated directly on data. On the other hand, the overall pattern appears very similar, with the same antagonistic variation in geometric components. (TIF) [file pcbi.1013416.s004.tif]

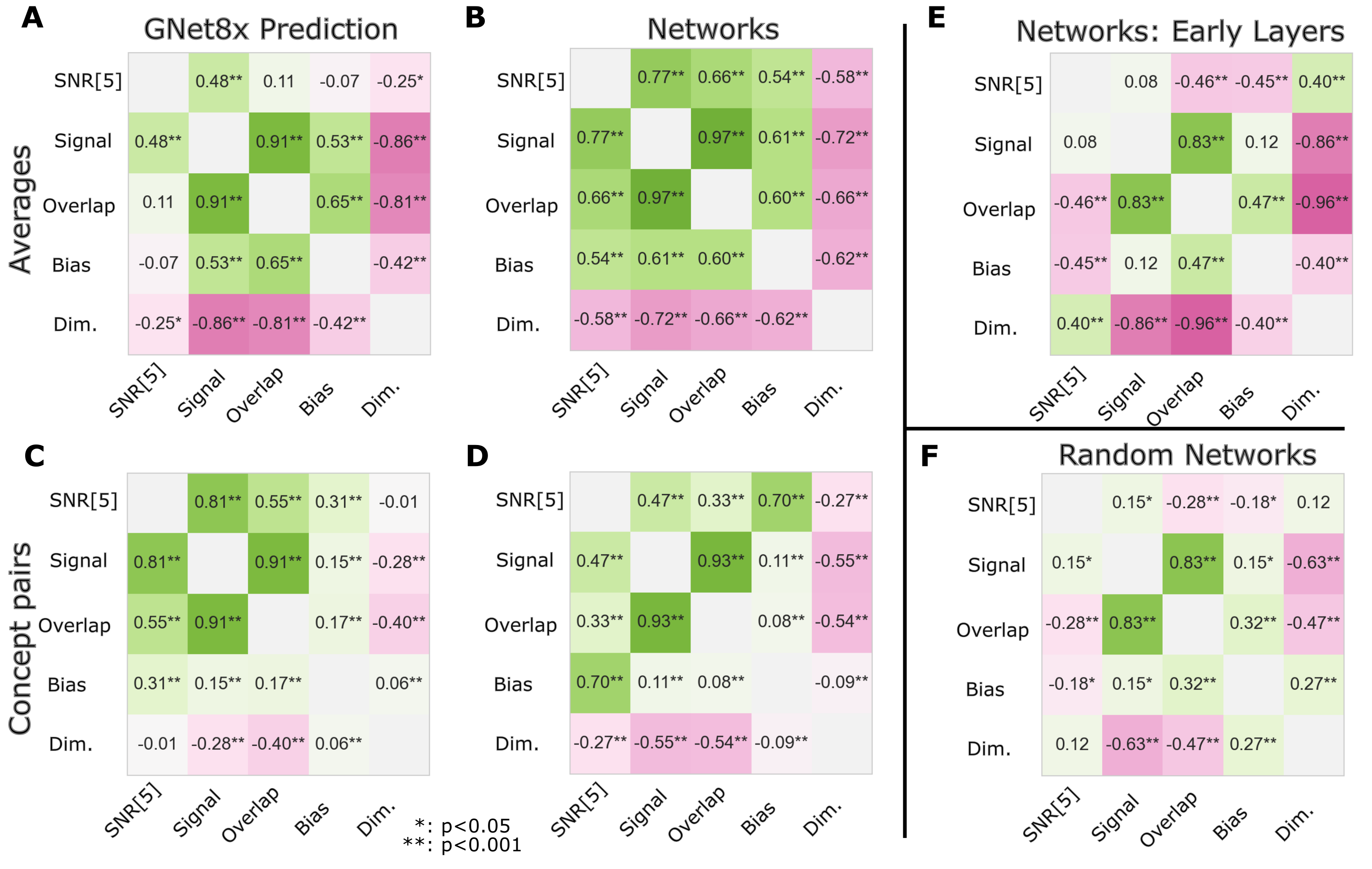

Supplement: S5 Fig — Correlations of the average of the estimates for A) brains B) and networks. Correlations for all concept pairs of the estimates for C) brains D) and networks. E) Correlation of the average of the estimates for early layers (layers with fractional depth smaller than 0.5) only. F) Correlation of the average of the estimates for randomized networks. (TIF) [file pcbi.1013416.s005.tif]

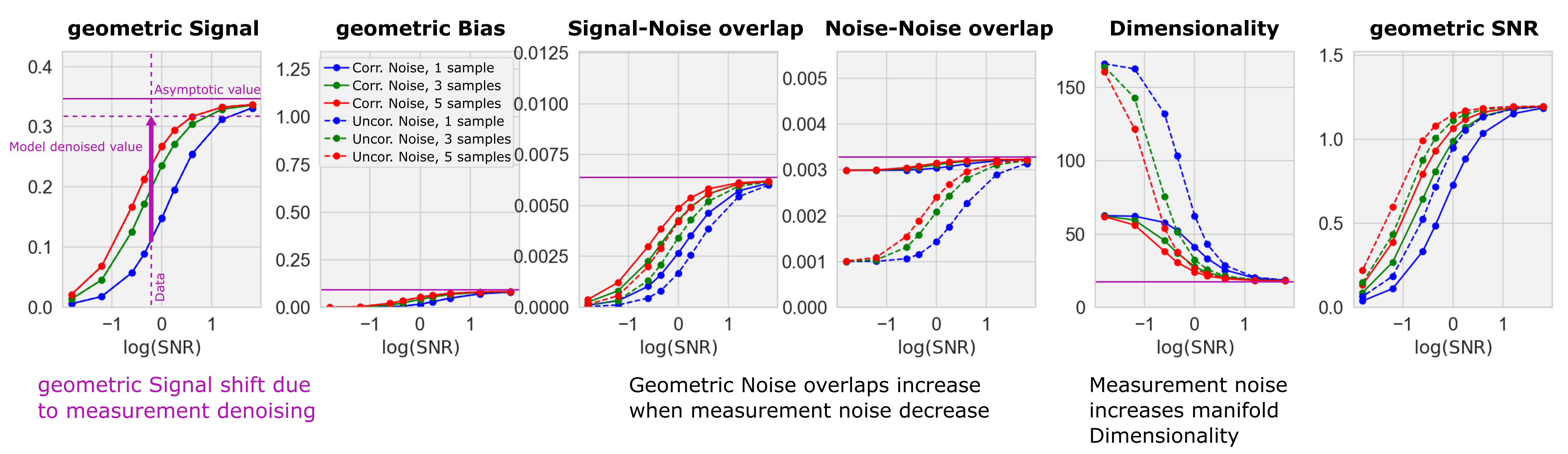

Supplement: S6 Fig — We simulated structured concept manifolds formed by sets of activity patterns with varying amounts of trial-to-trial variability, which we refer to here as “measurement noise”. We estimate geometric properties of the manifolds as a function of the measurement SNR (SNRm, x-axis), which is defined as the variance of the average activity across images, divided by the average variance of measurement noise. We consider both correlated (solid curves) and uncorrelated (dashed curves) measurement noise. We also show values of geometric properties inferred from trial-averaged activity with 1, 3 and 5 samples (colors) for each exemplar. The ‘true‘ (asymptotic) values of the geometric properties (solid magenta lines) are achieved when measurement noise tends to zero, i.e., when measurement SNR is large. Estimates also tend toward their asymptotic values as the number of samples available for trial-averaging increases. As shown in S4 Fig, we find that all estimates of geometric properties based on encoding model outputs are consistent with the ‘denoising‘ effect of an increased number of samples (this effect is illustrated schematically by the dashed magenta line in the leftmost panel). In other words, estimates of geometric properties based on encoding models yield higher estimated values for all properties except Dimensionality, which is exactly the effect of increasing the number of samples for trial averaging. Thus, the encoding model we use here impacts our estimates of geometric properties in the same direction as averaging across increased numbers of samples. (TIF) [file pcbi.1013416.s006.tif]

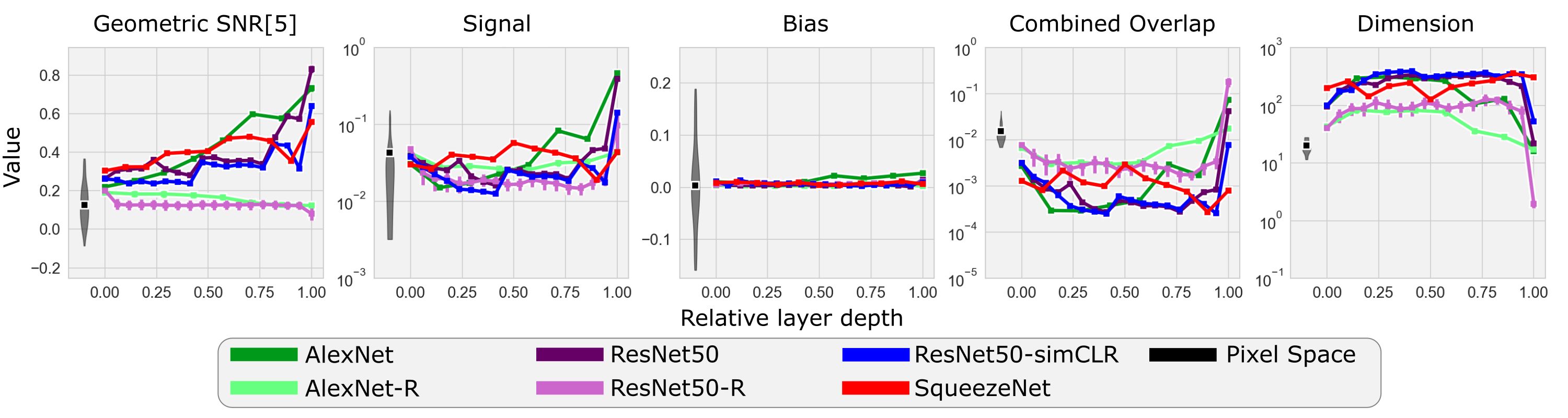

Supplement: S7 Fig — Two additional network geometries have been estimated: ResNet50 trained with contrastive learning (trained weights were obtained from https://github.com/AndrewAtanov/simclr-pytorch) and SqueezeNet, a denser architecture (50x less parameters) with similar performance as AlexNet (trained weights obtained from torchvision’s model zoo https://pytorch.org/vision/main/models.html). Compare to Fig 5. (TIF) [file pcbi.1013416.s007.tif]
